# Supplementary material for: Hot spot prediction in protein-protein interactions by an ensemble system
Source: BMC Syst Biol. 2018 Dec 31;12(Suppl 9):132. doi: 10.1186/s12918-018-0665-8 (PMC6311905; doi:10.1186/s12918-018-0665-8)
Supplement: Supplementary file 2 — All relevant MATLAB code. A simple Matlab implement of our predictor. (ZIP 19,742 kb) [file 12918_2018_665_MOESM2_ESM.zip › Additional file 2/Read ASA/ASA website/dbMPIKT/5E6PB.html]

 NetSurfP - Protein Surface Accessibility and Secondary Structure Predictions

  

|  |  |  |
| --- | --- | --- |
|  |  | NetSurfP - Protein Surface Accessibility and Secondary Structure Predictions  Technical University of Denmark |

  


---

```
# For publication of results, please cite:
# A generic method for assignment of reliability scores applied to solvent accessibility predictions.
# Bent Petersen, Thomas Nordahl Petersen, Pernille Andersen, Morten Nielsen and Claus Lundegaard
# BMC Structural Biology 2009, 9:51 doi:10.1186/1472-6807-9-51
#
# Column 1: Class assignment - B for buried or E for Exposed - Threshold: 25% exposure, but not based on RSA
# Column 2: Amino acid
# Column 3: Sequence name
# Column 4: Amino acid number
# Column 5: Relative Surface Accessibility - RSA
# Column 6: Absolute Surface Accessibility
# Column 7: Z-fit score for RSA prediction
# Column 8: Probability for Alpha-Helix
# Column 9: Probability for Beta-strand
# Column 10: Probability for Coil
E G  5E6PB                  1    0.839  66.053  -1.686   0.003   0.003   0.994
E P  5E6PB                  2    0.567  80.443  -1.208   0.018   0.019   0.964
E H  5E6PB                  3    0.589 107.194  -2.099   0.018   0.047   0.935
E M  5E6PB                  4    0.680 136.108  -1.820   0.018   0.047   0.935
E G  5E6PB                  5    0.618  48.652  -0.586   0.018   0.047   0.935
E L  5E6PB                  6    0.386  70.603  -0.552   0.018   0.088   0.893
E V  5E6PB                  7    0.447  68.673   0.126   0.004   0.420   0.576
E Q  5E6PB                  8    0.489  87.353   0.786   0.004   0.616   0.381
E R  5E6PB                  9    0.258  59.196   1.227   0.001   0.900   0.099
E C  5E6PB                 10    0.311  43.608   1.209   0.001   0.959   0.040
B V  5E6PB                 11    0.032   4.934   0.389   0.001   0.959   0.040
E I  5E6PB                 12    0.316  58.423   1.639   0.000   0.983   0.017
B I  5E6PB                 13    0.032   5.902   0.438   0.001   0.959   0.040
E Q  5E6PB                 14    0.406  72.422   1.370   0.001   0.959   0.040
E K  5E6PB                 15    0.323  66.441   0.656   0.004   0.514   0.481
E D  5E6PB                 16    0.656  94.558   0.157   0.005   0.015   0.979
E Q  5E6PB                 17    0.848 151.506  -0.813   0.003   0.003   0.994
E H  5E6PB                 18    0.639 116.234  -0.381   0.004   0.138   0.858
E G  5E6PB                 19    0.259  20.375  -1.571   0.005   0.262   0.733
B F  5E6PB                 20    0.125  25.087   0.741   0.004   0.616   0.381
B G  5E6PB                 21    0.065   5.131  -1.914   0.002   0.816   0.182
B F  5E6PB                 22    0.043   8.650   0.172   0.001   0.900   0.099
B T  5E6PB                 23    0.186  25.826   0.667   0.001   0.959   0.040
B V  5E6PB                 24    0.042   6.471   0.269   0.001   0.959   0.040
E S  5E6PB                 25    0.251  29.441   0.871   0.001   0.900   0.099
E G  5E6PB                 26    0.414  32.590  -1.912   0.004   0.197   0.799
E D  5E6PB                 27    0.685  98.752  -1.754   0.005   0.015   0.979
E R  5E6PB                 28    0.459 104.997  -0.198   0.005   0.045   0.951
B I  5E6PB                 29    0.262  48.544  -0.836   0.005   0.262   0.733
B V  5E6PB                 30    0.067  10.313   0.787   0.002   0.816   0.182
B L  5E6PB                 31    0.114  20.947   1.335   0.006   0.962   0.032
B V  5E6PB                 32    0.032   4.872   0.400   0.006   0.962   0.032
B Q  5E6PB                 33    0.207  37.060   0.961   0.011   0.918   0.071
E S  5E6PB                 34    0.303  35.453   1.092   0.018   0.846   0.136
B V  5E6PB                 35    0.046   7.055   0.593   0.004   0.616   0.381
E R  5E6PB                 36    0.348  79.600   1.007   0.004   0.197   0.799
E P  5E6PB                 37    0.675  95.825  -0.014   0.005   0.045   0.951
E G  5E6PB                 38    0.644  50.651  -1.672   0.016   0.005   0.979
B G  5E6PB                 39    0.130  10.231  -1.479   0.109   0.005   0.886
E A  5E6PB                 40    0.313  34.526   0.150   0.430   0.016   0.555
B A  5E6PB                 41    0.090   9.896  -1.770   0.455   0.046   0.498
E M  5E6PB                 42    0.442  88.504  -0.236   0.455   0.046   0.498
E K  5E6PB                 43    0.514 105.730   0.477   0.339   0.016   0.645
E A  5E6PB                 44    0.301  33.137  -0.947   0.181   0.016   0.803
E G  5E6PB                 45    0.427  33.629   0.012   0.058   0.017   0.925
B V  5E6PB                 46    0.048   7.347   0.603   0.053   0.043   0.903
E K  5E6PB                 47    0.340  69.917   1.315   0.052   0.084   0.864
E E  5E6PB                 48    0.463  80.816   0.466   0.052   0.084   0.864
E G  5E6PB                 49    0.331  26.034  -0.542   0.018   0.047   0.935
B D  5E6PB                 50    0.087  12.594  -0.639   0.004   0.138   0.858
B R  5E6PB                 51    0.186  42.548   1.043   0.011   0.918   0.071
B I  5E6PB                 52    0.033   6.087   0.643   0.001   0.959   0.040
B I  5E6PB                 53    0.077  14.245   1.351   0.001   0.959   0.040
B K  5E6PB                 54    0.226  46.529   0.486   0.001   0.959   0.040
B V  5E6PB                 55    0.042   6.440   0.371   0.001   0.900   0.099
B N  5E6PB                 56    0.237  34.711   0.134   0.005   0.262   0.733
E G  5E6PB                 57    0.587  46.165  -0.862   0.005   0.015   0.979
E T  5E6PB                 58    0.441  61.097   0.992   0.022   0.552   0.426
E M  5E6PB                 59    0.441  88.204   0.777   0.004   0.616   0.381
B V  5E6PB                 60    0.061   9.345  -0.101   0.020   0.205   0.775
E T  5E6PB                 61    0.523  72.540   0.876   0.018   0.047   0.935
E N  5E6PB                 62    0.667  97.663   0.436   0.018   0.019   0.964
B S  5E6PB                 63    0.139  16.338   1.738   0.115   0.016   0.868
E S  5E6PB                 64    0.410  48.017   1.317   0.053   0.043   0.903
B H  5E6PB                 65    0.106  19.354   1.042   0.970   0.001   0.030
E L  5E6PB                 66    0.592 108.377   1.546   0.970   0.001   0.030
E E  5E6PB                 67    0.472  82.528   1.947   0.970   0.001   0.030
B V  5E6PB                 68    0.021   3.166   1.196   0.970   0.001   0.030
B V  5E6PB                 69    0.126  19.305   1.504   0.970   0.001   0.030
E K  5E6PB                 70    0.596 122.515   1.863   0.970   0.001   0.030
B L  5E6PB                 71    0.173  31.695   1.256   0.970   0.001   0.030
B I  5E6PB                 72    0.041   7.529   0.375   0.923   0.002   0.076
E K  5E6PB                 73    0.454  93.408   1.361   0.782   0.003   0.216
E S  5E6PB                 74    0.651  76.344   0.590   0.406   0.004   0.590
E G  5E6PB                 75    0.403  31.708  -0.678   0.016   0.005   0.979
E A  5E6PB                 76    0.675  74.330  -0.729   0.005   0.015   0.979
E Y  5E6PB                 77    0.518 110.632   1.162   0.005   0.262   0.733
B V  5E6PB                 78    0.148  22.717   0.882   0.001   0.900   0.099
E A  5E6PB                 79    0.305  33.589   1.412   0.001   0.959   0.040
B L  5E6PB                 80    0.104  19.097   0.908   0.001   0.959   0.040
B T  5E6PB                 81    0.155  21.485   1.565   0.001   0.959   0.040
B L  5E6PB                 82    0.066  12.103   0.229   0.001   0.959   0.040
E L  5E6PB                 83    0.275  50.444   0.434   0.001   0.959   0.040
E G  5E6PB                 84    0.351  27.616  -0.264   0.001   0.900   0.099
E S  5E6PB                 85    0.475  55.611   0.236   0.004   0.420   0.576
E S  5E6PB                 86    0.539  63.147  -0.921   0.004   0.085   0.910
E P  5E6PB                 87    0.610  86.545  -1.372   0.018   0.019   0.964
E S  5E6PB                 88    0.919 107.742  -1.136   0.000   0.000   0.999

Explain the output.  Go back.
```

---
